# Interprofessional Care Models for Pregnant and Early-Parenting Persons Who Use Substances: A Scoping Review

**DOI:** 10.5334/ijic.7589

**Published:** 2024-06-06

**Authors:** Kristen Gulbransen, Kellie Thiessen, Natalie Ford, Wanda Phillips Beck, Heather Watson, Patricia Gregory

**Affiliations:** 1 University of Manitoba, CA; 2 University of British Columbia, CA; 3 Red Deer Polytech, CA; 4 Max Rady College of Medicine, University of Manitoba, CA

**Keywords:** substance use, pregnancy, postpartum, birth, interprofessional care

## Abstract

**Introduction:**

Use of substances during pregnancy is a global health concern. Interprofessional care teams can provide an optimal care approach to engage individuals who use substances during the perinatal period. The purpose of this scoping review is to provide a comprehensive summation of published literature reporting on interprofessional care models for perinatal individuals who use substances.

**Methods:**

We conducted a systematic search for articles from health-related databases. The Preferred Reporting Items for Systematic Reviews for Scoping Reviews (PRISMA-ScR) was followed. Data were extracted and synthesized to identify the interprofessional care team roles, program and/or provider characteristics, and care outcomes of these models.

**Results:**

We screened 645 publications for full text eligibility. Eleven articles met full inclusion criteria and were summarized. Programs were built on co-location of services, partnership with other agencies, available group/peer support and approaches inclusive of cultural care, trauma informed care, and harm reduction principles.

**Discussion:**

There is growing evidence supporting integrated care models that are inclusive of relational care providers from multiple health care professions to achieve wraparound care.

**Conclusions:**

Many of the interprofessional care models studied have successfully blended social, primary, pregnancy, and addictions care. The success and sustainability of programs varies, and more work is needed to evaluate program and patient outcomes.

## Introduction

Substance use during the perinatal period is a global health care concern and can result in profound consequences over the continuum of pregnancy through postpartum [1,2]. Substance use is broadly defined as alcohol use and illicit substances like opioids and stimulants. Worldwide, the majority of deaths attributed to substance use are related to opioid use [3]. To positively influence maternal and neonatal outcomes for perinatal individuals who use substances, the standard of care should include a wraparound approach addressing psychosocial, medical, and basic needs of the person [2,4]. Ideally a care model for perinatal individuals who use substances would include an interprofessional collaborative care team [5]. Health care providers that work collectively to meet the needs of a community and provide wraparound care improve patient care, particularly for patients with complex and/or chronic conditions [4,6,7].

Health care providers and international health organizations have identified care and treatment for substance use in pregnancy as a priority as it continues to impact maternal mortality and morbidity [4,8]. Pregnant individuals who use substances are recommended to intersect with various health care providers and the health care system throughout the continuum of pregnancy, birth, and postpartum [8]. Interactions with health care providers can leave lasting impressions on patients and families; those that are judgmental, and stigmatizing may last even longer [9]. Many clients enter pregnancy with feelings of guilt, shame, and uncertainty when they have used or use substances [10]. This may impede access to health care services due to a fear of punitive and legalistic consequences [11]. The interprofessional care team is well positioned to collaborate with perinatal individuals who use substances in efforts to improve maternal and neonatal outcomes and establish a safe trusting relationship within the existing health care system. An interprofessional care team is defined as a group of individuals from different professions that share knowledge with each other to enable effective collaboration and improve health outcomes [6].

## Background

Pregnancy can be a pivotal time for individuals who use substances to make life changes [2,12]. Many pregnant individuals who use substances stop using, decrease use, or initiate opioid agonist therapies when they learn they are pregnant [5]. Addiction is a complex disease involving changes to the thinking processes in the brain and often rooted in trauma and stress [13]. There is no single factor that determines if an individual will become addicted or not. Regardless of current substance use all deserve access to nonjudgmental health care; access to prenatal care during pregnancy is an influential precursor for improved birth outcomes [12,14]. Perinatal experts from North America and Australia have emphasized the benefits of an interprofessional care team inclusive of perinatal and addictions experts for prenatal through postpartum individuals who use substances as best practice [5,14,15,16,17]. Bringing multiple providers together has been recognized to increase engagement and actionable care services, improve communication among providers, and decrease traumatization or stigmatization.

Substance use during pregnancy does not exist in isolation and is often accompanied by adverse circumstances; these may include medical conditions like HIV, hepatitis, liver and cardiac diseases, poverty, gender violence, psychiatric comorbidity, polysubstance use, nutritional deficiencies, inadequate health care, and stress necessitating programs that provide care a multitude of needs are required [11,18,19]. Based on the potential complications and diverse care requirements for individuals who use substances, it is evident that throughout the continuum of pregnancy, birth, and postpartum care models require specialized services with multiple providers and programming attuned to their needs.

Programs in the United States and Canada dedicated to perinatal individuals who use substances began emerging in large urban areas in the early 1990’s as the substance cocaine was gaining popularity [1,21,22]. Substance use trends in North America include polysubstance, opioid, and methamphetamine use [3,12,15] among pregnant individuals who use substances. Prenatal care coupled with substance use treatment is the preferable approach to improve birth outcomes [14,15,16]. Opioid agonist treatment (OAT) programs to improve perinatal outcomes and retention of maternal guardianship for those that use opioids emerged in the early 2000’s in North America [14,15,16]. Despite the existence of programs and OAT therapy being available, barriers for accessing services still exist, these primarily include the threat of custody loss or child apprehension and stigmatization from the care provider [11,23,24,25]. As a result, substance use during pregnancy is one of the most missed and undertreated diagnoses during the perinatal period of care [14,16].

Nathoo et al. has described best practices for programs that offer care for pregnant individuals who use substances based on a review of four exemplar Canadian programs (Maxxine Wright Place, Healthy Empowered Resilient Pregnancy Program, HerWay, and Manito Ikwe Kagiike) [26]. The best practices elements include: (1) engagement and outreach, (2) harm reduction, (3) cultural safety (4) supporting mother and child, and (5) partnerships. The opportunity to have several team members comfortable to incorporate best practices in effort to care plan and/or provide crisis management can be invaluable [17]. There is an identified need for comprehensive best practice guidelines that are solution-focused, and delineate a safe, dignified, and compassionate care approach [15].

A gap in the literature is a summation of the existing interprofessional care models along with identification of the roles and characteristics of the interprofessional care team and program outcomes. Determining what interprofessional care models exist for perinatal individuals who use substances is key to guide the development and sustainability of programs. The purpose of the scoping review is to (1) provide a comprehensive summation of published literature reporting on interprofessional care models for pregnant through postpartum individuals who use substances, (2) identify the roles and characteristics of the interprofessional care team providers and programs that provide effective care for pregnant through postpartum individuals who use substances, and (3) identify interprofessional program team members, patient and program outcomes, care model and provider characteristics, philosophies, and goals emerging from these interprofessional care models.

## Methods

We conducted a scoping review of the literature to identify care models to develop, sustain, and improve care for perinatal individuals who use substances and early-parenting families who use substances. Scoping reviews address more complex and exploratory research questions, which includes identifying key concepts and gaps in the evidence [27]. The Arksey and O’Malley 5-stage framework for conducting scoping reviews guided the research process along with the updated recommendations from Levac et al. [28]. The five steps include: (1) Develop a research question, (2) Search for relevant material, (3) Define study selection, (4) Chart the data, and (5) Collate, summarize, and report results that are organized in tables according to research questions. The primary aim of this scoping review was to produce the first synthesis of interprofessional care models for pregnant, birthing, and postpartum families who use substances based on the peer reviewed literature describing the interprofessional roles, characteristics, and patient and/or program outcomes. The scoping review protocol is registered with Open Science Framework [29].

### Study Selection Criteria

**Figure 1:**
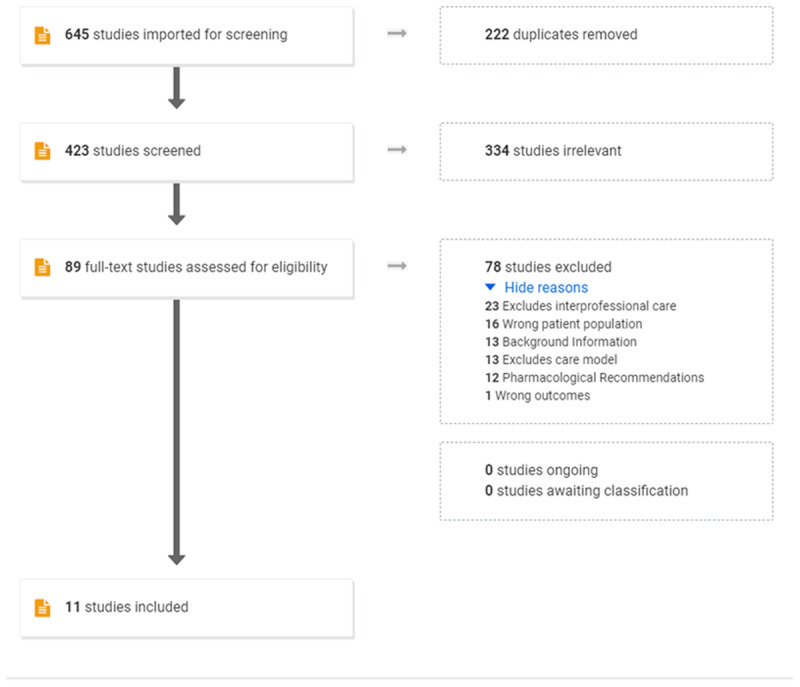
Preferred reporting items for systematic reviews and meta-analyses (PRISMA-ScR)–Scoping Review Extension flow diagram [32].

The Preferred Reporting Items for Systematic Reviews and Meta-Analyses (PRISMA- ScR) extension for scoping review requirements by Tricco et al. [30] was completed. The PRISMA diagram details the flow of study selection through the different phases of the scoping review (Figure 1). Screening of articles was conducted in two phases: title and abstract and then full text review. We used an iterative process to discuss and determine a consistent approach to answer the research questions [28]. We involved an interprofessional research team in the scoping review representing nursing, midwifery, and obstetrics. All review steps involved two or more reviewers.

Studies eligible for inclusion included peer-reviewed primary research reports, systematic reviews, and program websites or publications from credible databases and organizations. The following data was extracted: authors; year and type of study/publication; objectives; country of origin; study population and sample size; identification of roles on the interprofessional care team; description of characteristics of health care providers and programs; and program and patient outcomes associated with the interprofessional care team caring for pregnant, birthing, and early-parenting patients who use substances. Eligible studies included those that identified the interprofessional care team that provided care throughout the continuum of pregnancy, birth, and postpartum for persons who used substances. Exclusion criteria were animal studies and duplicates, studies that did not have an interprofessional care team involved in the care, did not provide care prenatal services through postpartum, did not discuss a care model, and studies that were limited to a pharmacological approach. We did not pre-define the types of outcomes such as the roles on the interprofessional care team and descriptions of the provider characteristics.

### Data Sources and Searches

The search strategy was designed with guidance from a professional medical librarian. The searches were then executed by the principal investigator (KG). All citations were managed in the EndNote reference management system. We conducted a systematic search for articles and reports from a wide range of electronic databases. Backward citation chasing also occurred using Scopus and Google scholar to identify potentially relevant literature. Search terms related to these three concepts were used: *substance use, perinatal, and interprofessional care*. Each concept included both subject headings (e.g. MeSH) and keywords, and was combined with Boolean operators (AND, OR) to produce a systematic search. These included the following health-related platforms and databases: MEDLINE (OVID); CINAHL with Fulltext (EBSCO); PsycINFO (OVID); Embase (OVID); the Web of Science (Clarivate), including Social Science Citation Index, Conference Proceedings Citation Index – Social Science & Humanities; and Social Services Abstract. The search did not have any limits set. Initial searches of two databases (CINAHL and Medline) were conducted to identify other potentially relevant keywords and subject headings. Following the initial search, the database strategies were finalized, and translated to all identified resources. Additionally, the references of full text documents were reviewed to identify any additional papers.

### Data Collection Procedures

Citations were saved and deduplicated in EndNote, using the Bramer method [31]. Citations were then screened using Covidence by KG and NF. All references selected for inclusion were organized in a shared folder amongst the team (KG, NF, & KT) on Covidence. Accurate citation counts at each stage were recorded through the PRISMA flowchart– see Figure 1 [30].

## Findings

We (KG & NF) screened 423 articles leading to full text review of 89 articles for our scoping review. They were primarily from North America, Europe, and Australia. They included published research, systematic reviews, best practice guidelines, commentaries, and special reports. Several of the published articles were informative for background information as they reported on the barriers to care, and the global concern related to the rise in substance use. We followed exclusion criteria and articles that did not include interprofessional care, did not include the perinatal population, did not have a care model, and/or were limited to providing pharmacological recommendations were removed. Key articles on the topic of substance use during the perinatal period were retained for background information.

The reviewers reached 100% agreement on the inclusion of the 11 articles for the final summary of this scoping review. Data from the articles were extracted using the Covidence extraction tool by KG and NF. The extraction data tool was modified in Covidence to follow PRISMA-ScR guidelines and collect additional data to answer the research objectives. Key findings from the 11 included articles, which answered the scoping review research questions, are summarized in Table 1. The final 11 articles were selected because they reported on the interprofessional care models, interprofessional care team, and/or care provider characteristics and program outcomes.

**Table 1:** Interprofessional Care Delivery Programs for pregnant through postpartum individuals who use substances.


INTERPROFESSIONAL PROGRAM, COUNTRY, & PUBLICATION DATE	PROGRAM TEAM MEMBERS	DESCRIPTION OF STUDY & DATA COLLECTION METHOD	STUDY PARTICIPANTS	PROGRAM AND PATIENT OUTCOME FINDINGS

Collaborative Outreach and Adaptable Care at Hallmark Health (COACHH) program. United States 2019. [33].	Nurse practitioner, social worker, executive director, community health worker.	Qualitative. Interviews.	COACHH program team members (n = 40).	High prenatal engagement. Initiating and maintaining opioid use disorder treatment. Increased mother-infant togetherness (custody, bonding). Improved life planning outcomes (secure housing).

Eight multiservice wraparound programs. Canada. 2021. [35].	Varied among sites but included: Obstetrician, midwife, nurse, nurse practitioner, social worker, addictions counsellor, family physician, psychiatry, case manager, art therapist, music therapist, dental hygienist & cultural liaison.	Mixed Methods. Survey & Interviews.	Service partners (n = 60) and program staff (n = 108) from 8 different programs.	Wraparound care improved access to services, health equity, and addressed social determinants of health. Partnerships increased providers understanding of clients’ experiences, challenges, and impacts of trauma and interconnected issues related to substance use. Increased integration of Indigenous cultural connections. Improved mother-child connection and child welfare outcomes.

Center for Addiction and Pregnancy (CAP) co-location program. United States. 1996. [20].	Obstetrician, pediatrician, midwife, addictions counsellor, lactation consultant,	Program retrospective report. Chart review.	CAP program patients (n = 100).	Increased prenatal care attendance at appointments. Decreased preterm deliveries. Decreased neonatal intensive care unit admissions.

TIDES collaborative care program. United States. 2021. [35].	Obstetrician, peer support, addictions counsellor, family physician, psychiatry, spiritual care.	Evaluation report. Chart review.	Patients from TIDES (n = 57 pregnant).	73% reporting no opioid use. 100% had full custody of their newborns.

Comprehensive care approach model. United States. 2022. [36].	Obstetrician, pediatrician, social worker, addictions counsellor, psychiatry, psychology, case manager, anesthesiology.	Best Practice Guideline. Commentary.	NA	Integration of the interdisciplinary approach is best practice to improve maternal outcomes, patient engagement, and maternal and neonatal outcomes.

Multidisciplinary clinic offering integrated, holistic care. United Kingdom. 2003. [37].	Obstetrician, pediatrician, midwife, nurse.	Program report. Chart Review.	Patients from the integrated clinic (n = 146).	Increased prenatal attendance. Clients reported stabilization and reduction of substance use. Safer substance use (not sharing needles).

The Women and Newborn Drugs and Alcohol Services (WANDAS) program. 2016. Australia. [38].	Obstetrician, pediatrician, midwife, social worker, addictions counsellor, psychiatry.	Program report. Chart Review.	Patients from the WANDAS program (n = 354).	90% attendance rate at appointments. mean gestation of 38 weeks and mean birth weight of 3000 grams. 2% admitted to the nursery for neonatal abstinence syndrome. 26 babies were placed into care by Child Protection and Family Support.

Toronto Centre for Substance Use in Pregnancy (T-Cup) program. Canada. 2011. [39].	Obstetrician, pediatrician, nurse, social worker, addictions counsellor, family physician, case manager, anesthesiologist.	Retrospective Study. Chart Review.	Patients from the T-Cup program(n = 121).	High compliance rate with prenatal care attendance. Most reported reduction in a variety of substances use categories. Significant differences were found among those who presented earlier in their pregnancies. Neonatal outcomes were satisfactory and approximately 75% of newborns were discharged with their mothers.

Wraparound Programs. Canada. 2020. [5].	Varied between sites but included: Obstetrician, midwife, nurse, nurse practitioner, social worker, addictions counsellor, family physician, psychiatry, case manager, art therapist, music therapist, dental hygienist & cultural liaison.	Mixed Methods. Interview and Survey Data.	Service partners (n = 60) and program staff (n = 108) from 8 different programs.	Resulted in better access for clients to health and social care, addressing trauma, and improved child welfare outcomes. Increased access to women’s mental health services, access for children’s health assessments and referrals, access to cultural programming, increased prenatal support and attendance at appointments, increased connection with peers for parenting programs.

Integrated Care Models. Canada & United States. 2018. [40].	Varied between sites but included: Obstetrician, pediatrician, midwife, nurse, nurse practitioner, addictions counsellor, family physician, psychiatry, peer support, psychology, pharmacist, case manager, patient navigator, childcare, pharmacist, occupational therapist, nutritionist, resource coordinator.	Qualitative Phenomenological. Interviews.	Program leaders from across North America (n = 23).	Families require frequent, supportive visits. Billable services can restrain this. Provider program champions are important but interprofessional care can still remain siloed. Addressing dual needs (newborn and parents) can be difficult if caregivers continue substance use.

Comprehensive Care Program. Canada. 1984. [41].	Obstetrician, pediatrician, addictions counsellor, psychology, child development educators.	Evaluation report. Chart Review.	Prenatal patients (n = 219) and Pre-pregnancy obstetrical patients (n = 191).	In four-fifths of the cases there were at least 5 prenatal visits. The dose of methadone at delivery remained stable. 5.2% had premature rupture of membranes, and obstetrical complications were absent in nearly two thirds of the cases. Newborn gestational age and birthweight were improved over outcomes for those that receive little or no prenatal care.


### Article Overview

We categorized the articles by design, including qualitative (n = 2), evaluation reports/program overviews (n = 7), best practice guideline (n = 1), and a retrospective study (n = 1). The published studies were from Australia (n = 1), Canada (n = 4), United States (n = 5), Canada and United States (n = 1), and the United Kingdom (n = 1). Most (n = 10) of the articles reported on patient outcomes related to their programs. These mostly included prenatal attendance, improved maternal health outcomes related to their substance use, mother-baby togetherness, and neonatal health outcomes. Table 1 provides an overview of all eleven articles selected, the program team members, a brief description of the study, study participants, and the reported program and patient outcomes.

### Summation of Interprofessional Care Models

#### Patient Enrollment and Continuity of Care within Programs

We reviewed the articles for patient enrollment criteria for each program and if there were referral processes. Enrollment and referral processes are important for patient accessibility and decision makers as they plan programs and identify costs. The inclusion criteria for pregnant individuals who use substances to participate in the reported programs during the prenatal period varied from substance abstinence [35] to program attendance that endorsed a harm reduction approach [5,34,37,41]. The TIDES program had 97% of their patients enter the program voluntarily and attributed the voluntary participation to the success of the program [35]. Programs such as the Collaborative Outreach and Adaptable Care at Hallmark Health (COACHH) program identified that there are challenges enrolling patients and measuring their program outcomes [33]. The pathway by which patients were engaged by or accepted into the other programs was not available.

Continuity of care for perinatal individuals that spans the continuum of pregnancy, birth, and postpartum is ideal to develop trusting relationships between patient and provider. We identified 6 programs that reported Provider continuity of care across the continuum inclusive of the birth period. The Center for Addiction and Pregnancy (CAP) program provided 24-hour midwifery coverage for the birth experience [20]. Oversight of labour and delivery coverage was not detailed by the other 5 programs. The CAP program was the only program that reported the inclusion of residential care for 5–7 days [20]. The residential care in the CAP program was followed by outpatient programming that included group education on obstetrical care, substance use, occupational therapy, family, relapse prevention, family planning, parenting, and lactation.

We determined that variances existed in length of services for postpartum care provided by the eleven programs analyzed in this scoping review. The care interval ranged from 6 weeks to 18 months postpartum. The WANDA’s program providers reported that they link patients to appropriate follow up care in the community before they discharge patients at 3 months postpartum [38]. The importance of a handoff to other care providers postpartum was likewise emphasized by some of the program leaders interviewed in Schiff and colleagues qualitative research study [40].

#### Co-location of Services

Co-location of services is an attempt to address the barriers of access to services and promote consultation and care from different providers in one site. All eleven publications reported varying multiple services offered in the same physical space. The identified benefits of co-location of services included decreased barriers to access and care providers being able to meet and discuss care planning [20,34,41]. Study participants in the programs reviewed identified the significant benefits of having basic needs of food, clothing, housing, and care for women’s health, substance use, trauma, child welfare, and/or children’s health services co-located as impactful to their pregnancy and postpartum health and care experiences [5,33,40]. Co-location of services can facilitate the wraparound approach for pregnant individuals who use substances focusing on social determinants of health, with provision of primary, prenatal, perinatal, and mental health services [5].

Most of the studies reviewed also discussed the establishment and importance of partnerships with community agencies located outside of their programs. Emphasis was placed on the importance of trusting partnerships and agreements when a recommended prenatal or postpartum service is not co-located [34,35]. McKinney and colleagues identified the importance of the interprofessional care teams’ interface and advocacy with the legal system in keeping families together [36]. Rutman and colleagues acknowledged that despite the importance and need of partnerships with housing, income assistance, Indigenous cultural programming, infant development, and legal services not all programs have these partnerships developed [5].

### Care Model Approaches

Exploration of care model approaches was conducted. Many of the interprofessional care models have successfully blended social, primary care, pregnancy care, and addictions care. All eleven publications referred to the importance of collaboration among care providers, prevention of child/infant removals and incorporating trauma informed and harm reduction principles. The Women and Newborn Drugs and Alcohol Services (WANDAS) program philosophy explicitly stated that they embed trauma informed approaches in all care interactions and assessment tools [38]. The harm reduction approach in the Toronto Centre for Substance Use in Pregnancy (T-Cup) program was integrated into the program to engage and retain pregnant individuals who use substances in care [39]. A few programs in the Co-Creating Evidence study reported incorporation of cultural care including Baby Welcoming ceremonies, spending time with Elders, traditional teachings, and participating in drum programs [5]. Group and peer support was offered in several of the programs however these authors did not describe what this looked like within their program [5,35]. Patients that experienced peer support described it as beneficial to develop friendships and be on the same road [5]. Table 2 outlines the care model approaches from each of the publications.

**Table 2:** Care Model Approaches.


STUDY	CO-LOCATION OF SERVICES	CULTURAL CARE	TRAUMA INFORMED	HARM REDUCTION	PARTNERSHIP WITH OTHER AGENCIES	GROUP/PEER SUPPORT

Collaborative Outreach and Adaptable Care at Hallmark Health (COACHH) [33].					**✓**	

Eight multiservice wraparound programs [34].	**✓**	**✓**	**✓**	**✓**	**✓**	**✓**

Center for Addiction and Pregnancy (CAP) [20].	**✓**					**✓**

TIDES collaborative care program. [35].	**✓**				**✓**	**✓**

Comprehensive care approach model. [36].			**✓**			

Multidisciplinary clinic. [37]	**✓**			**✓**		

The Women and Newborn Drugs and Alcohol Services (WANDAS) program [38].			**✓**	**✓**	**✓**	

Toronto Centre for Substance Use in Pregnancy (T-Cup) [39].	**✓**			**✓**	**✓**	

Eight multiservice wraparound programs. [5].	**✓**	**✓**	**✓**	**✓**	**✓**	**✓**

Integrated Care Models [40].					**✓**	

Comprehensive Care Program [41].	**✓**			**✓**		**✓**


### Roles and Characteristics of the Interprofessional Care Team

#### Interprofessional Care Team Roles

The interprofessional care teams consisted of 4 to 15 members in differing roles. The obstetrician was represented on all eleven of the interprofessional care teams in the publications reviewed. McKinney and colleagues described the obstetrician’s role as the provider responsible for coordinating care and ensuring appropriate referral to other care [36]. Additional team members identified in the programs reviewed included midwifery, nurse practitioner, patient navigator, pharmacy, nutritionist, childcare providers, dental hygienist, cultural liaison, art therapist, music therapist, and spiritual care provider. The importance of multidisciplinary team meetings was emphasized by a few of the authors to enhance coordinated care [35,36]. North American program leaders for perinatal individuals who use substances (n = 23) were interviewed in Schiff et al.’s phenomenological study [40]. They reported on the immense potential for furthering integrated care as a necessity, highlighting the requirements for changes in infrastructure, staffing, and loosening of the bonds of siloed maternal, infant, and addictions care in many settings [40]. Pregnant individuals who choose who they interact with on the interprofessional care team reported the positive impact related to patient experience and their autonomy [5,35].

#### Interprofessional Care Provider Characteristics

Many of the programs in the eleven articles indicated they were built on non-judgmental care, trusting relationships, and relational care. However, these characteristics are not well described with coinciding actionable approaches. McKinney and colleagues refer to a non-stigmatizing approach being necessary to promoting better outcomes with child welfare [36]. Mitchell et al. described the non-judgemental attitude as important to give the clients increased confidence and self-esteem, allowing questions to be asked, and being inviting to make decisions [37]. Shared-decision making was central to two of the programs (COACHH & T-CUP) as clients benefit when they decide what care providers, they need to be successful in the program [33,39]. Respect and positivity were other characteristics identified and not further described. Since it is well reported that significant barriers for pregnant individuals who use substances to engage or return for care are stigmatization, judgment, and fear the care provider characteristics identified make sense. Table 3 identifies the care characteristics of the program providers.

**Table 3:** Reported Care Provider Characteristics.


STUDY	NON-JUDGMENTAL	TRUST	RESPECT	SHARED DECISION MAKING	RELATIONAL

Collaborative Outreach and Adaptable Care at Hallmark Health (COACHH) [33].	**✓**	**✓**	**✓**	**✓**	**✓**

Eight multiservice wraparound programs [34].	**✓**	**✓**			**✓**

Center for Addiction and Pregnancy (CAP) [20].	None reported.				

TIDES collaborative care program [35].		**✓**			

Comprehensive care approach model [36].					**✓**

Multidisciplinary clinic [37].	**✓**				

The Women and Newborn Drugs and Alcohol Services (WANDAS) program [38].	**✓**	**✓**			

Toronto Centre for Substance Use in Pregnancy (T-Cup) [39].				**✓**	

Eight multiservice wraparound programs. [5].	**✓**	**✓**			**✓**

Integrated Care Models [40].		**✓**			**✓**

Comprehensive Care Program [41].	None reported.				


### Patient and Program Outcomes

#### Improved Maternal and Neonatal Outcomes

Overall, the programs reported beneficial outcomes when pregnant individuals who use substances engaged in specialized care with an interprofessional care team. Major findings included increased prenatal engagement, decreased substance use, and increased mother-baby togetherness/custody (see Table 1). Qualitative data from Mitchell and colleagues’ study reported on patients signals of change such as decreasing substance use and safer substance use [37]. Patients of the *TIDES* program and *T-CUP* program self-reported on abstinent opioid use during the postpartum period of the programs [35,39].

Many of the publications reported on the improved neonatal outcomes related to decreased preterm deliveries and decreased neonatal intensive care admissions [20,35,38,39]. The success of this was correlated with care providers from their programs fostering the newborn and mother relationship and advocating to keep women and their newborns together. The TIDES program emerged when North Carolina was spotlighted for opioid use and significant concerns with child placement in foster care [35]. The TIDES program was designed to avoid foster care placement and 100% of program participants (n = 57) had full custody of their newborns following completion of the program. The COACHH program reported that clients in their program had positive interactions with the Department of Children and Families and secure housing [33]. There is a lack of long-term outcomes for patients reported in the reviewed literature.

#### Discussion

Given the rise of substance use throughout the perinatal care continuum there is a need to have specialized programs staffed with interprofessional care providers. The importance of multi-sector, multi-service wraparound programming and partnership approach is evident amongst the literature reviewed. When there is a wide range of services provided and pregnant individuals who use substances can choose what services and care providers they interact with, a person and women centered approach is honored [5]. Studies reviewed in this scoping review were inclusive of an obstetrician, however, many settings may not have obstetrical resources. Perinatal expertise can be provided by other primary care providers such as midwifery and family practice physicians. The existing programs highlight positive maternal and neonatal outcomes promoting the need for them to scale and spread across the health care systems. When the services are offered and co-located, this results in pregnant individuals who use substances having increased engagement with the health care system.

Despite the number of growing programs and interest in program development for perinatal individuals who use substances, attention to a low barrier referral process into programs is required. Overcoming complex health care systems and limiters such as abstinence to promote successful referral and engagement must be addressed [36]. Partnership between care providers and agencies to promote care engagement is essential to prevent missed care opportunities that improve maternal and newborn outcomes [26,36]; the interprofessional care programs reviewed highlighted the increased engagement during the prenatal period. With a commitment to meeting the needs of pregnant through postpartum individuals who use substances, program leaders need to evaluate what services should be offered, how to foster relationships among the care team and with external community partners. A decision about who will be employed on the interprofessional care team also needs to be considered. Resource constraints along with the high demand for substance use programs and detoxification programs are likely a reality in many settings throughout the world. Given the current context and strains in many healthcare systems utilizing existing care models and evidence-based approaches rather than creating new ones is sensible.

Policy and program planners need to advocate for resources and share the positive impacts for the pregnant person, family, and community when an interprofessional care approach is integrated into the health care system. The discourse to obtaining non-judgmental care among pregnant individuals who use substances is challenging. Persons are often framed with the double stigma of being a substance user or “addict,” along with the perception as a parent-to-be lacking maternal commitment in the eyes of society [44]. Advocacy is required to reconsider that narrative, as substance use (or lack thereof) does not define parental capacity. Care providers working with pregnant individuals who use substances are in a precarious position as they navigate the patient-provider confidential relationship with the role of mandatory reporting for child abuse and neglect. Patients may perceive a dual loyalty; providers scope should be transparent with the patient before and after communication with child welfare [17]. Stigma associated with substance use or “addiction stigma” is thought to exacerbate the structural and individual level barriers to treatment and care [45]. Intersectional stigma exists among pregnant individuals who use substances; the stigma is a result of the dynamic interaction of multiple marginalized social identities [46]. Therefore, incorporating the components of trauma informed care and harm reduction are vital in facilitating safe access [4,17]. When organizations do not enact policies to provide nonjudgmental, trauma informed, and culturally grounded approaches, the result can lead to decreased prenatal care [5]. Cultural care inclusive of individuals perspectives align with a wraparound approach by viewing cultural engagement as healing [42,43]. When someone has experienced stigma and a negative health care interaction, they are less likely to engage in care, for any reason again [46]. The consequences of shame-based barriers such as stigmatization can be grave for both the parent and newborn. The programs that identified the importance of a nonjudgmental approach did not provide a nuanced approach to enact being nonjudgmental.

Providers who care for pregnant individuals who use substances had a philosophy of care that demonstrated commitment to the population. Based on the literature reviewed, there is limited description of how patients perceive care provider characteristics in the health care setting. Further exploration of the impact of approaches taken by the care providers that are nonjudgmental, trusting, relational, respectful, and positive could provide insight into provider training. Care providers have reported that they have not received specialized or adequate education and training in substance use [14]. Training for healthcare professionals is still in the early adoption stages when it comes to care for persons with substance use disorders [36]. The programs analyzed in this scoping review did not discuss the education and training that their interprofessional care team possessed to work in the programs. Nathoo et al. acknowledges that many Canadian programs have taken their own initiative to provide training for health professionals on substance use and pregnancy [26] but measurements on impact related to patient outcomes and providers knowledge and attitudes is not reported.

#### Gaps in the Literature

Research in this area is already limited by under-reporting likely related to the stigmas related to substance use during pregnancy and inconsistent screening. Quantifying outcomes directly related to the programs for substance use among pregnant persons has been a challenge [33,39]. This scoping review was limited to programs published in the literature for this population and the search terms’ limits. Many of the publications reported on the perspectives of program employees [40] and a volunteer sampling approach was taken with patients [5,34]. Studies have yet to identify the relative contribution of program components (care provider make up) and provider characteristics that contribute to positive perinatal outcomes.

Much of the published literature provides overviews of the programs without statistically significant or comparative evidence. Marcellus et al. has highlighted the need for evaluation indicators to move away from using abstinence as a measure of success [47]. Ordean and Kahan’s retrospective study identified that there are many confounding factors in their data analysis including baseline severity of addiction and level of functioning [39]. Finding the right quantitative outcome measurements is noted as difficult; instead, success has been measured in qualitative terms for many programs.

Qualitative interviewing with program providers and participants is a guided approach to facilitate sharing of experiences and perspectives. It is possible that participants will not have shared all aspects and perspectives related to their experiences. The existing literature and the publication reviewed in the scoping review highlighted the importance of care provider characteristics as non-judgmental, trusting, and relational from the provider perspective. However, a significant gap in the literature is the patients’ perspectives on what provider characteristics are important and what they look like in practice. The nuances of these relationships remain unknown. Patient perspectives and patient engaged research are key to the success of programs. Thus, patient perspectives need to be further explored by researchers.

## Conclusion

We explored the existing interprofessional care models that have been published and affirmed that the interprofessional care approach is best practice for perinatal individuals who use substances. We found that encounters between pregnant individuals who use substances and a dedicated interprofessional care team have positive outcomes. The interprofessional care team that integrates care promotes collaboration with the patient at the center. Further deploying the already successful strategies that are included in many of programs is a logical first step as the health care system evolves to provide further programming and care for pregnant individuals who use substances. Wraparound programming has been remarkable in providing blended social and primary care services for pregnant individuals who use substances [5]. A trauma-informed, culturally sensitive, and harm reduction approach influences retention in the programs.

Decreasing stigmatization as a barrier to care matters; every encounter between a health care provider and person who is substance involved during the prenatal through the early-parenting periods is an opportunity to build a trusting relationship. An environment where the interprofessional care team can effectively collaborate with external community partners is important to increase the mother and newborn togetherness and to reduce unnecessary costs, trauma, and involvement of the legal system. Fostering ways for trusting partnerships during the transitions between and within settings is key to promoting pregnant individuals who use substances satisfaction with the health care system. The interprofessional care team providers and those that provide peer support within the programs have the unique opportunity to contribute to improved social, maternal, and neonatal outcomes for perinatal individuals who use substances. Perhaps most importantly, the patients themselves have an important leadership role to play in defining the care goals of these endeavours, particularly when they are engaged as experts early in program conception.
